# Renal injury, biomarkers, and myositis, an understudied aspect of disease: prospective study in the MyoCite cohort

**DOI:** 10.3389/fmed.2023.1127657

**Published:** 2023-06-22

**Authors:** Edoardo Conticini, R. Naveen, Parikshit Sen, Mantabya Singh, Upendra Rathore, Anamika Kumari Anuja, Mohit Kumar Rai, Brijesh Yadav, Narayan Prasad, Vikas Agarwal, Latika Gupta

**Affiliations:** ^1^Rheumatology Unit, Department of Medicine, Surgery and Neurosciences, University of Siena, Siena, Italy; ^2^Department of Clinical Immunology and Rheumatology, Sanjay Gandhi Post Graduate Institute of Medical Sciences, Lucknow, India; ^3^Maulana Azad Medical College, New Delhi, India; ^4^Department of Nephrology, Sanjay Gandhi Postgraduate Institute of Medical Sciences, Lucknow, India; ^5^Department of Rheumatology, Royal Wolverhampton Hospitals NHS Trust, Wolverhampton, United Kingdom; ^6^Division of Musculoskeletal and Dermatological Sciences, Centre for Musculoskeletal Research, School of Biological Sciences, The University of Manchester, Manchester, United Kingdom; ^7^Department of Rheumatology, City Hospital, Sandwell and West Birmingham Hospitals NHS Trust, Birmingham, United Kingdom

**Keywords:** myositis, India, renal, biomarker, damage

## Abstract

**Introduction:**

The mechanisms leading to chronic kidney disease (CKD) in patients with idiopathic inflammatory myopathies (IIMs) are poorly understood. We assessed the prevalence of subclinical renal injury in patients with IIMs, through elevation in biomarker levels of tubular injury and fibrosis (NGAL, KIM1, Activin A, CD163, and Cys-c), and assessed differences between subtypes of IIMs, and the effect of disease activity and duration.

**Materials and methods:**

Clinical data, core set measures, sera and urine were prospectively collected from all patients enrolled in the MyoCite cohort from 2017 to 2021. Twenty healthy subjects (HC) and 16 patients with acute kidney injury (AKI) were included as controls. Baseline and follow up data for IIMs were included. Enzyme-linked immunosorbent assay (ELISA) was used to measure urine NGAL (Human Lipocalin-2/NGAL Duoset ELISA, Cat no: DY1757), KIM1 (Human TIM-1/KIM 1/HAVCR Duoset ELISA, Cat.no: DY1750B), Activin A (Human Activin A Duoset ELISA, Cat no: DY338), CD163 (Human CD163 Duoset ELISA,Cat no: DY1607-05), and Cys-c (Human Cystatin C Duoset ELISA, Cat. no.: DY1196) levels, while eGFR (unit mL/min/1.73 m2) was calculated by the Cockcroft-Gault formula and CKD-EPI formula.

**Results:**

Analysis of 201 visits of 110 adult patients with IIMs indicated higher normalized biomarker levels compared to HCs, and comparable to patients with AKI, with the exception of NGAL, which was higher in the AKI group. Notably 72 (49%) patients with IIMs had eGFR<90; the levels of the 5 biomarkers were comparable between active and inactive IIMs, and different subtypes of IIMs. Similarly, a poor correlation between urine biomarker levels and core set measures of activity and damage was found. Changes in biomarker levels on follow-up did not correlate with eGFR changes.

**Discussion:**

This exploratory analysis of urinary biomarkers identified low eGFR and elevated biomarkers of CKD in nearly half of the patients with IIMs, comparable to patients with AKI and higher than HCs, indicative of potential renal damage in IIMs that may have a lead to complications in other systems.

## Introduction

Inflammatory idiopathic myopathies (IIMs) are a heterogeneous group of disorders primarily affecting the striate muscles, encompassing disease subtypes including dermatomyositis (DM), polymyositis (PM), anti-synthetase syndrome (ASS), cancer-associated myositis (CAM), inclusion-body myositis (IBM), and immune mediated necrotizing myopathy (IMNM) (1). In addition to striate muscles, these conditions often involve other organs or systems, with varying degrees of severity (2).

The kidneys and the urinary tract neither represent a common nor a typical target of disease activity, being usually spared from the inflammatory process. However, the massive release of creatine kinase (CK) and myoglobin during acute phases of inflammation has been observed to cause acute kidney injury (AKI) in nearly half of patients with rhabdomyolysis (3). A recent large French multicenter study reported that up to 23% of patients with IIMs may display a certain degree of renal involvement (4).

Though uncommon, the occurrence of this dreaded complication in patients with IIMs is not negligible. Regrettably, renal damage is often overlooked in current clinical practice. This is largely due to creatinine being by far the most common, and often the sole biomarker of renal function requested in clinical practice, which does not allow for the evaluation of renal injury and may lead to the overestimation of glomerular filtration rate (GFR) and overall renal function.

In recent years, several novel biomarkers of clinical and subclinical renal injury have been identified (5). These include neutrophil gelatinase-associated lipocalin (NGAL), Activin A, CD163, cystatin-C (Cys-c), and kidney injury molecule-1 (KIM-1). KIM-1 and NGAL are tubular injury biomarkers and are newly expressed in renal injury, hence referred to as *de novo*-synthesized biomarkers. NGAL is a 25-kDa protein of the lipocalin super family. It is a critical component of the innate immune response to bacterial infection and is expressed by immune cells, hepatocytes, and renal tubular cells in various disease states (6–8). Due to its specificity, the use of KIM-1 as a biomarker has substantially improved the diagnostic approach to acute kidney injury. The reference range in healthy individuals ranges from 0.00 to 398.60 pg./mL (9).

Activin A inhibits the regeneration of renal tubules following ischemic injury. It acts as a potent inducer of renal fibrosis and is implicated in the development of glomerulonephritis, lupus nephritis as well as acute kidney injury. In contrast to NGAL and KIM-1, however, Activin A and Cys-c are markers of glomerular damage or fibrosis (10). Finally, CD163, a marker of macrophage activation, strongly correlates with chronic damage, interstitial inflammation, and fibrosis (11).

These biomarkers correlate well with drug-induced and non-drug-induced subclinical renal injury, especially in the very early phases of the disease course. Among rheumatic disorders, these biomarkers have demonstrated predictive value for renal inflammation, tubular damage and utility in monitoring renal disease (12–14). However, data on their value in predicting renal injury in patients with IIMs is limited.

Thus, the primary objective of this study was to assess the prevalence of subclinical renal injury by elevated NGAL, KIM1, Activin A, CD163, and Cys-s levels in patients with IIMs. Secondary objectives included evaluating the differences in these biomarker levels between adults and children, different subsets of IIMs, and to compare their levels with disease activity and damage indices, to find if a correlation exists with disease duration. We also assessed the rate of GFR decline in patients with elevated biomarkers compared to those with normal levels.

## Materials and methods

Clinical data, core set measures, sera and urine were prospectively collected from all patients enrolled in the MyoCite cohort from 2017 to 2021. Twenty healthy individuals (HCs) and 16 patients affected by AKI (KDIGO definition) were included as a control group. For patients with IIMs, data from both baseline and follow-up visits within the observational period were included.

### MyoCite database

The MyoCite database includes prospectively collected data of Indian patients with myositis (diagnosis of IIMs made clinically by two rheumatologists) receiving treatment at the inpatient and outpatient clinics of the Department of Clinical Immunology and Rheumatology from 2017 to 2021 (15). The database includes detailed and standardized longitudinal clinical and laboratory data, with a matched biorepository, as part of a study approved by the local ininstitutional ethics committee. Adults and children enrolled in the Myocite cohort who initially presented. with possible myositis (as per two rheumatologists) between December 2017–March 2021 were further screened to only include subjects with a diagnosis of IIMs at baseline visit (as per 2017 ACR/EULAR classification criteria for DM/PM) for this study (16). We followed the STROBE guidelines while reporting the methods and results of our study (17). The various definitions used have been previously described (18).

### Types of IIMs

DM and PM were defined according to the ACR/EULAR criteria, as definite or probable (16). Overlap myositis was diagnosed in patients who fulfilled both the Bohan-Peter criteria and any single criterion for connective tissue disease (19–23). Anti-synthetase syndrome was defined by the presence of any three of 5 clinical features (fever, Raynaud’s phenomenon, arthritis, ILD, mechanic’s hand) and one of the ARS. CAM were defined as IIM with a malignancy diagnosed 3 years before or after the onset of myositis (24).

### Clinical details

Parameters collected included age, sex, definite diagnosis of IIMs, date of onset of symptoms, date of diagnosis, clinical features and outcomes, as well as core set measures (HAQ-DI, patient global assessment [PtGA], physician global assessment [PhGA], manual muscle test 8 [MMT8], muscle damage index [MDI], myositis disease activity assessment tool [MDAAT], muscle enzymes) (25). MDAAT > 1 (0–10) and MDI extent of severity score > 1 (0–38) were used to define active myositis and damage, respectively (25). MDAAT>1 was considered as “active” disease, while MDAAT ≤ 1 constituted “inactive” disease. The detailed specifications of the MyoCite cohort are previously published and available online (15).

### Antibody testing

Patients were evaluated for the presence of myositis specific (MSAs) or myositis associated antibodies (MAAs) by Line immunoassay (G4, Euro-Immune, Lubeck, Ger many), and those with 2+ or more were considered to be positive. The presence of Anti-Nuclear Antibodies (ANA) was tested using Immunofluorescence as say (IFA) using Hep-2010 cell line at a dilution of 1:100.

### Estimation of biomarkers

Enzyme-linked immunosorbent assay (ELISA) technique was used to measure urine NGAL, Activin A, KIM, CysC and CD163 levels. A clean, morning midstream urine sample (5 mL) was collected in a sterile test tube and centrifuged at 5,000 rpm for 15 min. The supernatant was transferred to an Eppendorf tube and stored at −80°C until assessment. An ELISA kit (R&D Systems, United States) was used as per manufacturer’s instructions (26).

### Urine protein and creatinine estimation

A venous blood sample (5 mL) was drawn undercomplete aseptic precautions after overnight fasting. The blood was centrifuged at 5,000 rpm for 10 min at room temperature, and serum was separated for routine hematology. Biochemistry, urinalysis, and urine protein measurements were performed as per study protocols. An automated blood-cell analyzer (BC-5380; Mindray, Shenzhen, People’s Republic of China) was used for routine hematology testing, and an automated clinical biochemistry analyzer (Cobas C 311; Roche-Hitachi, Tokyo, Japan) was used for measurement of blood urea nitrogen, creatinine, uric acid, serum lipids, and albumin (26). Patients with signs or symptoms of urinary tract infections were excluded beforehand.

### GFR estimation

eGFR (unit mL/min/1.73 m2) was calculated by the Cockcroft-Gault (CG) and CKD-EPI formulae (EPI) (27).

### Statistical analysis

Non-parametric tests were employed for analysis. Descriptive statistics alone were performed for each au toan ti body sub group when the sample size in any analytic group was less than 5. All data were expressed as mean ± standard deviation. Following as sess ment of data dis tri b u tion, univariate analysis was performed using the Student’s t-test or Mann Whitney U tests for con tinuous variables and Chi square test and Fisher’s ex act tests for categorical variables. One-way non parametric analysis of variance (ANOVA, Bartlett’s test) was used for comparisons between groups. Variables with more than 10% of the data missing were excluded from the analysis.

Pearson r test was employed to correlate urine biomarkers with core set outcome measures for disease activity and damage.

*p* value <0.05 was considered statistically significant, all reported values were 2-sided. IBM SPSS version 20 was used for analysis.

### Ethics

Ethical Approval was obtained from the local institutional ethics committee of Sanjay Gandhi Postgraduate Institute of Medical Sciences, Lucknow, India.

## Results

### Patients

**Figure 1:**
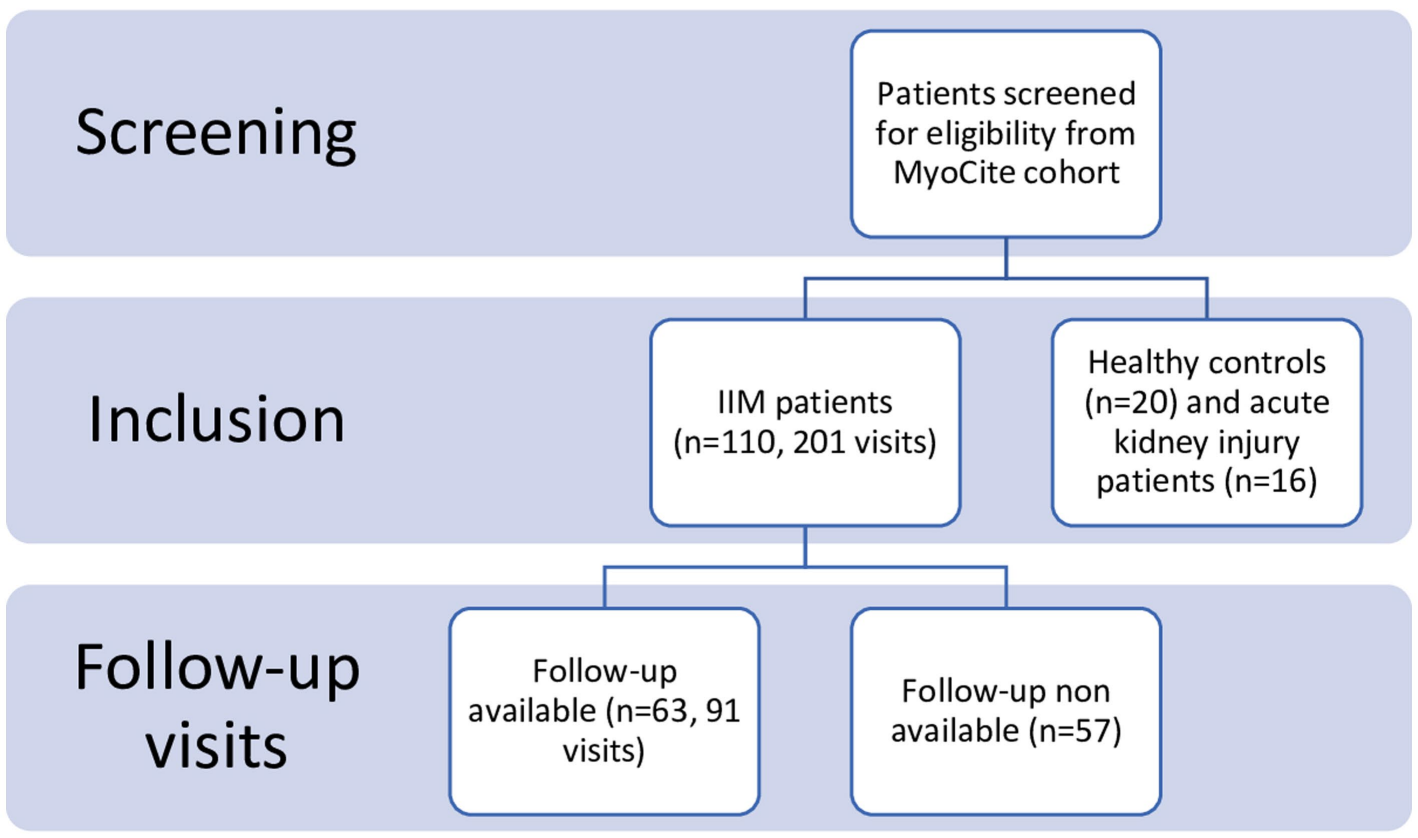
STROBE flowchart of baseline and study visits.

Two hundred and one visits of 110 adult patients (110 at baseline, 91 follow-up evaluations of 63 patients) were included in the analysis (Figure 1). Patients had a mean age of 41 (31.5–51) years, included 84 females and 26 males, and had a mean disease duration at baseline of 5 (3–13.5) months. The median follow-up duration was 16 (5-24) days. DM was the most prevalent IIM subtype (*n* = 47, 42%), with anti Ro52, followed by anti Jo-1 and anti Mi-2 being the most common antibodies. Nearly all patients (*n* = 103, 92%) suffered from muscle weakness, while skin and lung involvement was present in 63 (57.3%) and 41 (37.3%) patients, respectively.

In patients with IIMs, normalized urine biomarker levels were: NGAL/urine creatinine 316 ng/mg, KIM1/urine creatinine 1.23 ng/mg, Activin A/urine creatinine 83 ng/mg, CD63/urine creatinine 233 ng/mg, and Cys-c/urine creatinine 935 ng/mg. The median eGFR was 92 (70–112) (CG) and 107 (90–119) (EPI). Clinical and serological features, including urine biomarker levels, of patients are detailed in Table 1.

**Table 1:** Population characteristics of IIM patients.

Variables	Baseline (*n* = 110)	Follow-up (91 visits of 63 patients)
Age (years)	41 (31.5–51)	35 (28–48)
Females (*n*, %)	84, 0.76%	72, 79%
Disease duration (months)	5 (3–13.5)	6 (3–13)
**IIM subtypes**		
DM	47 (42.7)	46 (50)
OM	23 (20.9)	17 (18.5)
ASS	25 (22.7)	20 (21.7)
PM	11 (10)	5 (5.4)
CAM	4 (3.6)	3 (3.3)
**Clinical features**		
Muscle weakness	103 (92)	86 (93.5)
Any skin rash	63 (57.3)	55 (59.5)
Weight loss	57 (51)	69 (75)
Fever	54 (49.1)	44 (47.8)
Arthritis	45 (40.9)	33 (35.9)
ILD	41 (37.3)	31 (33.7)
RP	32 (29.1)	25 (27.2)
Malar rash	32 (29.1)	30 (32.6)
Mechanic’s hands	1 (0.9)	–
Dysphagia	21 (19.1)	15 (16.3)
Heliotrope rash	27 (24.5)	18 (19.6)
Gottron’s sign	22 (20)	17 (18.5)
Gottron papule	21 (19.1)	19 (20.7)
V sign	16 (14.5)	16 (17.4)
Periungual erythema	9 (8.2)	11 (12)
Shawl sign	10 (9.1)	8 (8.7)
Dysphonia	4 (3.6)	–
Cutaneous ulcerations	4 (3.6)	3 (3.3)
Calcinosis	1 (0.9)	1 (1.1)
**MSA/MAA**		
Anti Jo-1	11 (10)	10 (10.9)
Anti Mi-2	10 (9.1)	18 (19.6)
Anti SRP	4 (3.6)	3 (3.3)
Anti NXP2	3 (2.7)	5 (5.4)
Anti TIF1y	6 (5.5)	2 (2.2)
Anti MDA5	9 (8.2)	3 (3.3)
Anti SAE1	2 (1.8)	–
Anti Ro52	15 (13.6)	10 (10.9)
Anti Ku	4 (3.6)	2 (2.2)
Anti Pm Scl	3 (2.7)	–
Negative	43 (39.1)	38 (41.3)
**ANA patterns**		
Speckled	38 (34.5)	35 (38)
Negative	28 (25.5)	17 (18.5)
Homogenous	14 (12.7)	17 (18.5)
Nucleolar	10 (9.1)	4 (4.3)
Cytoplasmic	6 (5.5)	5 (5.4)
DFS	5 (4.5)	2 (2.2)
**Myositis core set measures**		
PtGA (VAS 0–10)	2 (0–4)	0 (0–2)
PhGA (VAS 0–10)	2 (0–1.50)	1 (0–2)
MDAAT (0–10)	0 (0–0.89)	0 (0–0.33)
SGOT (IU/L)	35 (24–65)	33 (25–53)
SGPT (IU/L)	35 (24–69)	34 (25–51)
CPK (IU/L)	105 (47–265)	103 (64–281)
LDH (IU/L)	474 (330–681)	329 (211–513)
MMT 8	77 (69–80)	79 (74–80)
MDA (VAS 0–10)	0 (0–2)	0 (0–3)
GDA (VAS 0–10)	0 (0–4)	0 (0–4)
HAQ-DI	0.69 (0–1.5)	0.125 (0–0.625)
MDI severity of damage (VAS 0–110)	8.5 (3.6–13.7)	1.5 (0–3)
**Biomarkers**		
Urine creatinine (mg/dl)	60 (35–106)	70 (40–107)
Urine NGAL (ng/mL)	155 (108–310)	202.5 (110–388)
Urine KIM1 (ng/dl)	85 (42–138)	79 (49–107)
Urine Activin (ng/mL)	51 (46–61)	48 (38–59)
Urine sCD163 (ng/mL)	132 (120–149)	127 (119–137)
Urine cystatin C (ng/mL)	462 (426–592)	600 (495–888)
NGAL/U. creatinine (ng/mg)	316 (164–684)	352 (140–704)
KIM1/ U. creatinine (ng/mg)	1.23 (0.58–2.46)	1 (1–2)
Activin/ U. creatinine (ng/mg)	83 (48–173)	68 (41–145)
CD 163/ U. creatinine (ng/mg)	233 (124–411)	180 (107–345)
Cystatin C/ U. creatinine (ng/mg)	935 (507–1,481)	925 (545–2,763)

DM, dermatomyositis; OM, overlap myositis; ASS, anti synthetase syndrome; ARS, aminoacyl tRNAse antibody; IBM, inclusion body myositis; PM, polymyositis; JDM, juvenile dermatomyositis; CAM, cancer associated myositis; ILD, interstitial lung disease; RP, Raynaud phenomenon; MSA, myositis specific antibodies; MSA, myositis associated antibodies; DFS, dense fine speckled; MDAAT, myositis disease activity assessment tool; MMT, manual muscle testing score; MDA VAS, muscle disease activity VAS; GDA VAS, global disease activity VAS; MDI, myositis damage index, HAQ DI, health assessment questionnaire disability index.

### Urine biomarkers in IIM compared with HC and AKI patients

Patients with IIMs had higher normalized urine biomarker levels compared to HCs (NGAL 327 vs. 184 *p* 0.024, KIM1 1.2 vs. 0.1 *p* < 0.001, Activin A 80 vs. 23 *p* < 0.001, CD163 202 vs. 34 *p* < 0.001, Cys-C 935 vs. 229 *p* < 0.001) (Table 2A; Figure 1). All biomarkers were equally elevated in patients with IIMs and AKI (KIM1 1.2 *VS* 1, Activin A 80 *VS* 72, CD163 193 *VS* 226, Cys-C 931 *VS* 1649), without any statistically significant differences, except normalized NGAL which was significantly more elevated in AKI patients (327 IIM vs. 937 AKI, *p* 0.009) (Table 2B). Seventy-two (49%) patients with IIMs had eGFR<90 (35% had 30–60, 11% had 15–30, 2% had <15 eGFR).

**Table 2:** (A) Urine NGAL, KIM-1 in IIM vs. HC.

(A)			
	IIM (*n* = 266)	HC (*n* = 20)	*p*
Urine creatinine (mg/dl)	65 (35–105)	57 (79–152)	0.436
Urine NGAL/creatinine ratio (ng/mg) (A)	327 (157–695)	184 (101–453)	0.024
Urine KIM-1/creatinine ratio (ng/mg) (B)	1.2 (0.6–1.9)	0.1 (0.05–0.3)	<0.001
Urine Activin A/U. creatinine (ng/mg) (C)	80 (46–156)	23 (9–36)	<0.001
Urine sCD163/U. creatinine (ng/mg) (D)	202 (112–387)	34 (14–68)	<0.001
Urine cystatin C/U. creatinine (ng/mg) (E)	935 (512–1925)	229 (82–363)	<0.001

(B) Urine NGAL, KIM-1 in IIM *vs* AKI (defined by KDIGO).			
	IIM (*N* = 201)	AKI (*N* = 16)	*p*
Urine NGAL/creatinine ratio (ng/mg)	327 (157–694)	937 (225–1,363)	0.009
Urine KIM-1/creatinine ratio (ng/mg)	1.2 (0.6–1.9)	1 (0.3–3.7)	0.848
Urine Activin/U. creatinine (ng/mg)	80 (46–155)	72 (37–187)	0.837
Urine sCD163/U. creatinine (ng/mg)	193 (112–369)	226 (113–580)	0.623
Urine cystatin C/U. creatinine (ng/mg)	931 (511–1915)	1,649 (767–2,412)	0.171

KIM1, kidney injury molecule 1; Neutrophil gelatinase associated lipocalcin; IIM, idiopathic inflammatory myopathy; MDAAT, myositis disease activity assessment tool.

### Urine biomarkers in active and inactive IIM and across different subtypes of IIM

**Figure 2:**
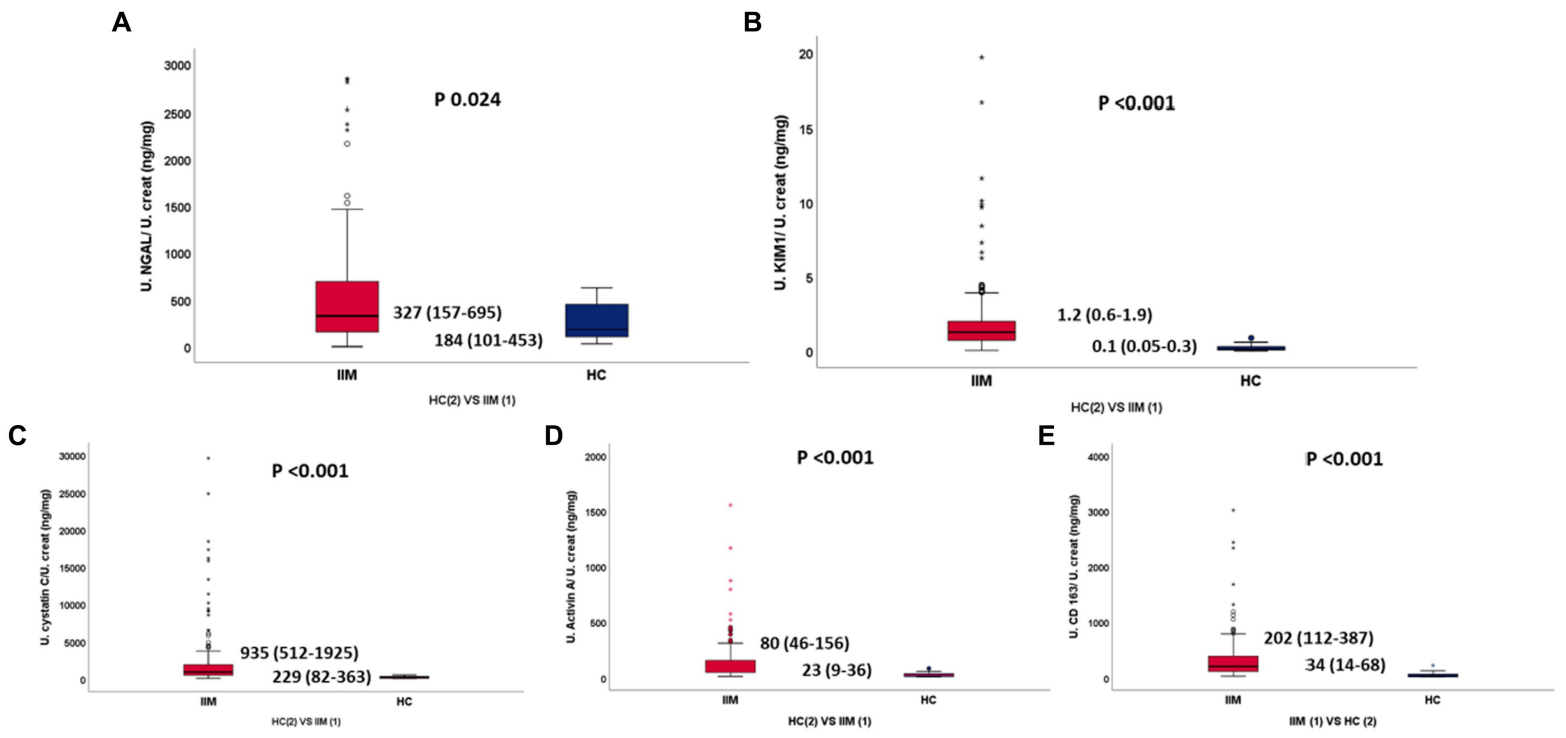
**(A–E)** Normalized urinary NGAL, KIM1, Cystatin C, Activin A, sCD163 between IIM and HC, respectively.

**Figure 3:**
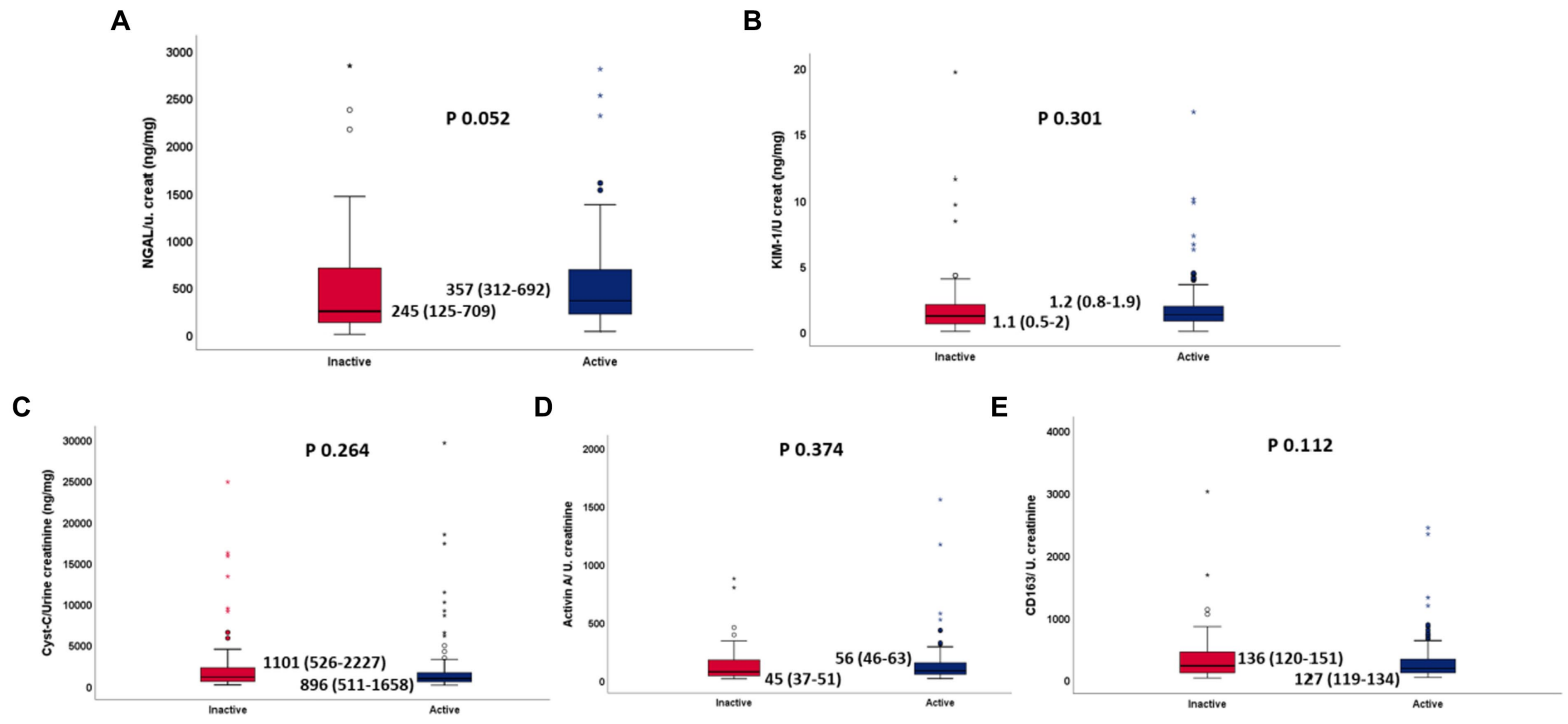
**(A–E)** Normalized urinary NGAL, KIM1, Cystatin C, Activin A, sCD163 between active and inactive IIM, respectively.

When patients were stratified according to disease activity, no statistically significant differences were found for all 5 biomarker levels (NGAL 357 vs. 245 *p* 0.052, KIM1 1.2 vs. 1.1 *p* 0.301, Activin A 72 vs. 82 *p* 0.374, CD63 181 vs. 224 *p* 0.112, Cys-c 896 vs. 1101 *p* 0.264) between patients with active (MDAAT >1) and inactive (MDAAT≤1) IIMs (Table 3; Figures 1, 2). A similar proportion of patients with active (90%) and inactive disease (88%) had elevated urine biomarkers. In ANOVA, no differences in urine biomarker levels were seen across different subtypes of IIM (Table 4; Figure 3).

**Table 3:** Urine NGAL, KIM-1 in active versus inactive IIM.

	Active IIM (MDAAT≥1) (*N* = 115)	Inactive IIM (MDAAT<1) (*N* = 76)	*p*
Urine creatinine (mg/dl)	70 (40–100)	60 (30–110)	0.587
Urine NGAL (ng/mL)	230 (116–391)	142 (99–277)	0.004
Urine KIM-1 (ng/dl)	88 (53–129)	69 (31–120)	0.033
Urine Activin (ng/mL)	56 (46–63)	45 (37–51)	<0.001
Urine sCD163 (ng/mL)	127 (119–134)	136 (120–151)	0.002
Urine Cystatin C (ng/mL)	514 (452–648)	507 (438–711)	0.968
Urine NGAL/creatinine ratio (ng/mg)	357 (212–692)	245 (125–709)	0.052
Urine KIM-1/creatinine ratio (ng/mg)	1.2 (0.8–1.9)	1.1 (0.5–2)	0.301
Urine Activin/U. creatinine (ng/mg)	72 (38–174)	82 (50–150)	0.374
Urine sCD163/U. creatinine (ng/mg)	181 (112–330)	224 (111–447)	0.112
Urine cystatin C/U. creatinine (ng/mg)	896 (511–1,658)	1,101 (526–2,227)	0.264

KIM1, kidney injury molecule 1; Neutrophil gelatinase associated lipocalcin; IIM, idiopathic inflammatory myopathy; MDAAT, myositis disease activity assessment tool.

**Table 4:** Urinary kidney injury biomarkers in subsets of IIM.

	ASS (*n* = 44)	DM (*n* = 91)	OM (*n* = 40)	PM (*n* = 16)	CAM (*n* = 7)	*p* (by ANOVA)
Urine creatinine (mg/dl)	62 (36–130)	60 (35–105)	70 (40–107)	72 (24–127)	60 (30–110)	0.561
Urine NGAL (ng/mL)	185 (108–405)	187 (103–298)	161 (111–347)	157 (113–294)	251 (55–445)	0.733
Urine KIM-1 (ng/dl)	66 (40–121)	85 (44–126)	79 (40–125)	96 (54–160)	70 (56–156)	0.557
Urine Activin (ng/mL)	50 (39–61)	52 (43–61)	48 (40–60)	50 (44–57)	45 (12–59)	0.554
Urine sCD163 (ng/mL)	126 (115–136)	126 (118–139)	134 (122–143)	140 (130–153)	131 (120–139)	0.065
Urine Cystatin C (ng/mL)						
Urine NGAL/creatinine ratio (ng/mg)	333 (159–718)	342 (177–752)	252 (136–690)	244 (150–469)	387 (249–698)	0.703
Urine KIM-1/creatinine ratio (ng/mg)	1.2 (0.5–1.8)	1.2 (0.7–2)	1.1 (0.7–1.8)	2 (0.5–3)	1.5 (0.7–2.2)	0.108
Urine Activin/U. creatinine (ng/mg)	76 (39–121)	85 (48–176)	63 (48–116)	77 (32–151)	94 (59–204)	0.531
Urine sCD163/U. creatinine (ng/mg)	191 (91–345)	214 (113–410)	190 (112–352)	172 (110–402)	272 (169–511)	0.326
Urine cystatin C/U. creatinine (ng/mg)	730 (401–1,433)	1,076 (616–2,271)	973 (528–1761)	708 (356–1960)	1,322 (738–4,431)	0.477

ASS, anti-synthetase syndrome; DM, dermatomyositis; OM, overlap myositis; PM, polymyositis; CAM, cancer associated myositis; KIM1, kidney injury molecule 1; Neutrophil gelatinase associated lipocalcin, *p* < 0.05 significant (ANOVA based on median).

### Correlation of biomarkers with core set measures

**Figure 4:**
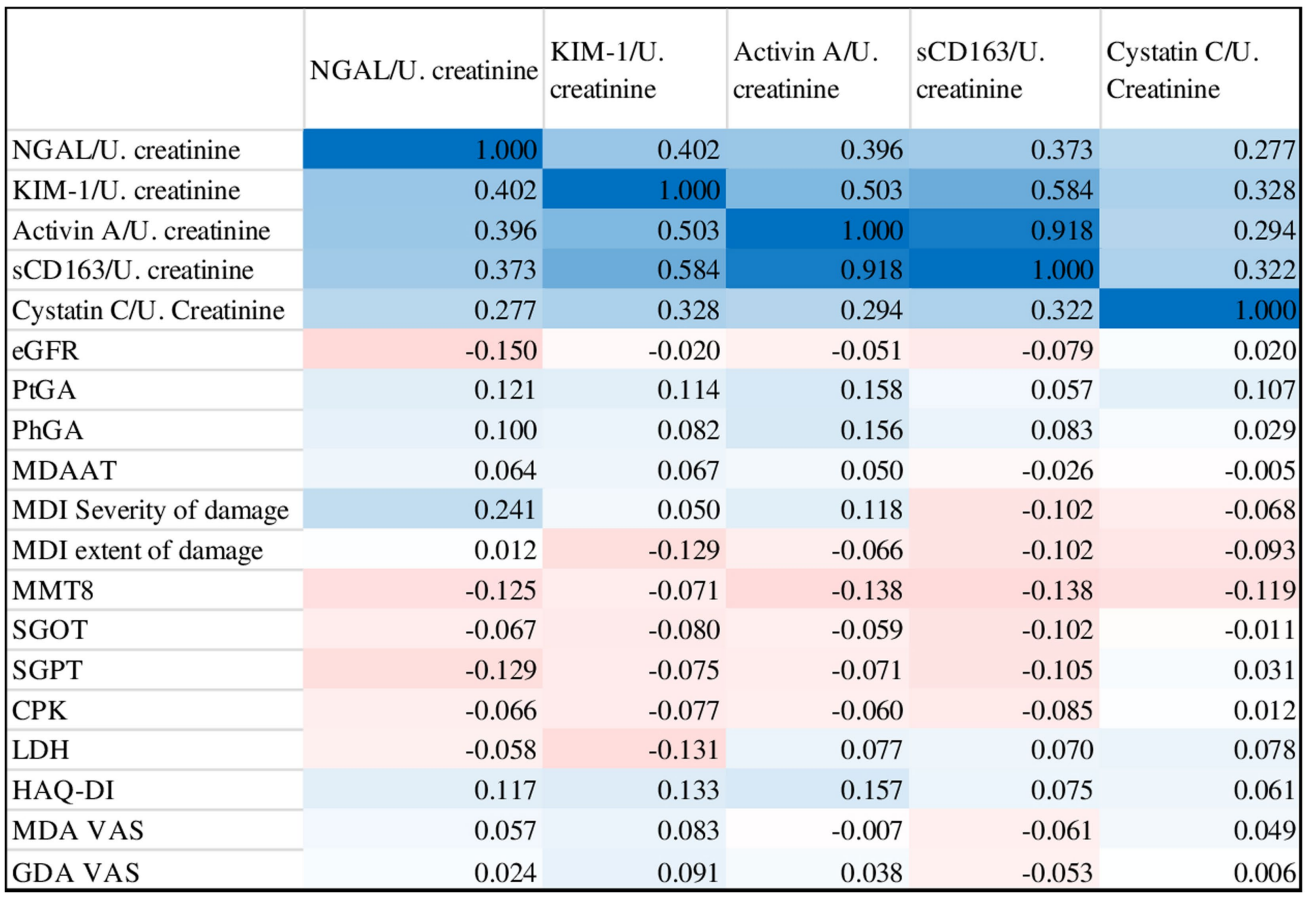
Correlation matrix of urinary biomarkers with myositis core set measures and other disease activity outcomes (blue indicates positive correlation, red indicates negative correlation).

A poor correlation was found between urine biomarker levels and core set measures. NGAL was positively correlated with MDI (r 0.241, *p* 0.01), while Activin A was positively with PtGA (r 0.158, *p* 0.024), PhGA (r 0.156, *p* 0.032) and HAQ-DI (r 0.157, *p* 0.03). However, no statistically significant correlation was found for the other biomarkers (Figure 4).

Conversely, an excellent, statistically significant, positive inter-correlation was found for all biomarkers.

Changes in the levels of these normalized biomarkers on follow-up did not correlate with changes in eGFR. Further sensitivity to change in disease activity could not be assessed as most patients did not have a change in disease activity on follow up.

## Discussion

Our exploratory analysis provides important insights into the prevalence and extent of CKD in patients with different subtypes of IIMs and differing disease activity. Increased levels of five biomarkers of CKD were observed in the majority of our subjects, and nearly half had eGFR<90.Notably these urinary biomarker levels were higher in patients with IIMs compared to HCs and were comparable to the patients with AKI. However, no correlation was found between the levels of these biomarkers with disease activity or subtype of IIM.

Although not considered a typical target of disease activity in IIMs, the kidneys may be directly or indirectly involved during the disease course in myositis. Clinical presentation may range from acute kidney injury to chronic glomerulonephritis. Acute kidney injury usually develops abruptly during acute phases of rhabdomyolysis in the background of severe muscle inflammation and has been widely explored and described in association with myoglobinuria (3), especially in patients with IMNM. Conversely, with the exception of crescentic glomerulonephritis, CKD has a far more insidious course, and though several mechanisms have been hypothesized, this condition has a different nature, and its mechanism is still poorly understood in patients with IIMs.

Our study not only found the occurrence of renal injury in a much higher percentage of patients with IIMs than previously reported (4), but also found the levels of 5 urine biomarkers as reliable indicators of renal injury comparable to individuals with diagnosed AKI. KIM1 and Activin A have been previously studied in AKI, AAV and SLE (10–13), as well as in healthy controls, in whom a reference range of (0.00–398.6 pg./mL) has been proposed for KIM1 (9). We observed comparable levels between subtypes of IIM, indicating a possible area for future exploration into potential pathogenetic mechanisms including myoglobin induced chronic smoldering tubular injury, or the downstream immunological impact of oxidative damage such as the activation of TGF beta pathways (28).

Kidney injury has not been extensively explored nor well described in all subtypes of IIMs from a clinical perspective. It is conceivable that myoglobinuria-mediated microtubular injury also may occur in other subtypes of IIMs in addition to patients with IMNM, which may possibly be asymptomatic and not require immediate intervention. In a French cohort, renal involvement (AKI and CKD) was seen in 23.3% of patients (4). The main cause of AKI was drug or myoglobinuria-induced acute tubular necrosis, and the outcomes of these patients were poor, with 81% progressing to CKD and 12.5% reaching end-stage renal disease. Thus, AKI and CKD may be frequent in patients with IIMs, with a peculiar pattern of acute vascular damage part of the spectrum of renal diseases associated with IIMs. Early detection of subclinical renal injury in these patients may be opportune for the early interventions to delay or even halt progression and reduce poor renal outcomes.

Proposed pathogenetic mechanisms of CKD in IIMs include tubular necrosis, as well as the chronic release of myoglobin and creatine-kinase by inflamed muscle. Recently, a possible role of autoimmunity has been speculated, in light of evidence of macrophage activation through the release of extracellular traps and complement factors, C3 and C5b-9 (29–31). As early as in the 1970s, Dyck et al. suggested the possible role of immune complexes consisting of myoglobin-anti-myoglobin antibodies in triggering glomerular injury, and Nishikai et al. documented the presence of anti-myoglobin antibodies in the sera of 22 of 31 patients with PM (32, 33). It is possible that the deposition of immune complexes may represent the first step of glomerular damage in most patients with IIMs, subsequently leading to complement deposition and leukocytic infiltration producing the observed pathological and clinical consequences. In addition to classical variants of IIM, patients with overlap myositis (OM) with SLE (lupus nephritis) and scleroderma may also have renal involvement. Of particular concern is the nephrotoxic effect of the prolonged use of many of the drugs used in the management of these conditions, which may further aggravate the disease associated renal injury.

Preliminary studies indicate that patients with PM usually present with mesangial proliferative GN, membranous nephropathy is more common among DM patients (34). OM usually presents with milder muscle disease, though in a previous study we found higher creatinine levels in these patients, potentially contributed by underlying renal disease in addition to higher muscle mass (15). We have also previously noted elevated levels of muscle biomarkers such as FABP3 in several patients with IIMs (35).

While the lack of correlation with the subtype of IIM is unsurprising, given that he prevalence and pathology of renal damage in different subtypes of IIMs is still poorly understood, the lack of correlation with disease activity has greater relevance. The preliminary design of our study warrants caution in drawing firm conclusions though it outlines an important agenda for further exploration. It may be possible that the current core set of measures for myositis do not adequately take renal involvement into account, and therefore the lack of correlation may be owing to an underestimation of disease activity (in certain subtypes of IIM), or even more plausibly, renal damage.

We may also suspect that, given the multifactorial causation of kidney injury in patients with IIMs, while the elevation of urine biomarkers in the active phase of disease may be due to the current disease activity, the increase of these biomarkers during the inactive disease phase may be due to cumulative renal damage or secondary to drug toxicity or treatment burden. It may also be plausible that the release of minor amounts of myoglobin over a long period of time may culminate in tubular injury and consequent renal damage. Due to the high morbidity and mortality associated with it, early assessment of renal injury is critical in the clinical management of IIM. However, since established markers such as serum creatinine overestimate renal function and may be erroneous due to loss of muscle mass specifically in IIM, urinary biomarkers may possibly be of better value as indicators of early renal injury before significant damage accrues.

Our study did have its limitations. We had a relatively low number of controls, which may have led to the underestimation of levels biomarkers levels in HCs. It was not possible to stratify our patients according to current and past treatment, due to the relatively high number of immunosuppressive drugs taken concomitantly or prescribed after the failure of first-line treatment. Thus, it was difficult to precisely triangulate the role of drug-induced kidney injury in our sample. We did not analyze electrolytes and urine microscopy, and due to the real-life design of our study, none of the patients underwent a renal biopsy. This was beyond the scope of the study, as renal involvement was not expected to start with. Thus, we were not able to distinguish between glomerular and tubular damage. However, given the absence of frank symptoms of active glomerulonephritis, it is likely most of our patients suffered from chronic tubular damage. Forth, we did not stratify our patients according to concomitant and previous treatments, therefore we were not able to assess whether any decline in renal function was secondary to drug toxicity.

Finally, the relatively short follow-up period and lack of disease activity variation during control visits did not allow the evaluation of the role of these biomarkers in the long-term decline of renal function. Our observations suggest that this is an important aspect to be considered while managing patients with myositis and is an area for further exploration in larger prospective cohort studies.

To conclude, our study found a high prevalence of renal injury in patients with IIMs and NGAL, KIM1, Activin A, CD163, and Cys-c as reliable biomarkers in its early diagnosis. Further studies are needed to better evaluate renal involvement in patients with IIMs and to accurately assess renal function in early stages of renal injury in these patients. Larger, long-term, multicenter studies should investigate different pathogenic mechanisms leading to CKD in these patients (including a possible iatrogenic role), as well as assess the implications of this condition on multiple systems, in terms of poor quality of life, accelerated atherosclerosis, and poor muscle function (36, 37). In this regard, recent evidence has displayed that myokine can in turn slow down kidney fibrosis, remarking the strict crosstalk existing between muscle and renal function (38).

## Data availability statement

The original contributions presented in the study are included in the article/supplementary material, further inquiries can be directed to the corresponding authors.

## Ethics statement

The studies involving human participants were reviewed and approved by Ethical Approval was obtained from the local institutional ethics committee of Sanjay Gandhi Postgraduate Institute of Medical Sciences, Lucknow, India. The patients/participants provided their written informed consent to participate in this study.

## Author contributions

LG: conceptualization and methodology. LG and RN: software, validation, and formal analysis. RN, AA, and MS: investigation. LG and VA: resources and supervision. LG, AA, RN, and MS: data curation. UR, EC, and RN: writing – original draft. EC, RN, PS, MS, UR, AA, MR, BY, NP, VA, and LG: writing – review and editing. LG and NP: visualization. All authors contributed to the article and approved the submitted version.

## Funding

This work was funded by APLAR research grant, Intramural funding, VA.

## Conflict of interest

The authors declare that the research was conducted in the absence of any commercial or financial relationships that could be construed as a potential conflict of interest.

## Publisher’s note

All claims expressed in this article are solely those of the authors and do not necessarily represent those of their affiliated organizations, or those of the publisher, the editors and the reviewers. Any product that may be evaluated in this article, or claim that may be made by its manufacturer, is not guaranteed or endorsed by the publisher.
